# Endotoxin modulates the electrophysiological characteristics of human embryonic stem cell‐differentiated cardiomyocytes

**DOI:** 10.1002/jcp.27251

**Published:** 2018-09-14

**Authors:** Shiji Wang, Yu Wang, Xin Guo, Yingji Li, Li Yan, Chengxi Wei, Ming Zhao

**Affiliations:** ^1^ First Hospital of Jilin University Changchun Jilin China; ^2^ Medicinal Chemistry and Pharmacology Institute, Inner Mongolia University for the Nationalities Tongliao Inner Mongolia China; ^3^ Affiliated Hospital of Inner Mongolia University for Nationalities Tongliao Inner Mongolia China; ^4^ Inner Mongolia Key Laboratory of Mongolian Medicine Pharmacology for Cardio‐Cerebral Vascular System Tongliao Inner Mongolia China

**Keywords:** arrhythmia, delayed rectifier potassium channel show activation current, lipopolysaccharide (LPS), L‐type calcium current, sodium current

## Abstract

Gram‐negative bacteria‐induced infections result in fever, arrhythmia, and even death. Lipopolysaccharide (LPS), a constituent of bacteria, leads to an inflammatory response under sepsis and increase arrhythmogenesis. This study analyzed the effects on human embryonic stem cell‐differentiated cardiomyocytes (HIPSC‐CMs) exposed to LPS. A whole cell patch clamp was used to record the action potential (AP) and ionic currents with or without different doses of LPS in HIPSC‐CMs. Compared with the control, a different dose (0.04, 0.2, 1, and 5 µg/ml) of LPS‐treated HIPSC‐CMs resulted in a longer AP duration. The IC50 of sodium channel current was 1.254 µg/ml, L‐type calcium channel current was 5 µg/ml, and *I_k_* channel currents were 1.254 µg/ml. LPS‐treated HIPSC‐CMs showed a lower sodium channel current, L‐type calcium channel current, and *I_k_* channel currents. Furthermore, the expressions of Nav1.5 were decreased, and L‐Ca, Kv11.1, and Kv7.1 were increased in LPS‐treated HIPSC‐CMs. LPS‐induced arrhythmogenesis was related to the electrophysiological characteristics of sodium channel current, L‐type calcium channel current, and *I_k_* channel currents. These results suggest a potential mechanism of cardiomyocyte injury in endotoxemia.

## 1 INTRODUCTION

Due to a large number of endotoxins released into the blood by gram‐negative bacteria, endotoxemia is the most common clinical systemic inflammatory response (Liu et al., 2009; Tang et al., 2010; Tang et al., 2011). Endotoxemia causes hemorrhages, necrosis of the kidneys, and myocardial dysfunction. Systemic inflammatory response syndrome is the main feature of endotoxemia. Due to the interaction between the severe infection and the hyperactive inflammatory response, progressive systemic organ failures (heart) may develop in the final stage (Cai et al., 2014). Lipopolysaccharide (LPS) is the main chemical component of endotoxin and initiates the host’s natural immune defense response (Nduka & Parrillo, 2011; Van Amersfoort, Van Berkel & Kuiper, 2003). Systemic LPS exposure (endotoxemia) has been linked to inflammation and immune activation in a range of pathologies (Palmer et al., 2016). Myocardial dysfunction is a common complication that significantly contributes to the mortality of sepsis in both patients and experimental models of LPS‐induced endotoxemia (Wang et al., 2015). However, the mechanism of cardiomyocyte injury due to endotoxemia remains poorly understood.

Isolated ventricular myocytes from the guinea‐pig heart have been used to analyze electrophysiological characteristics (Zhao et al., 2013). While HL‐1 cardiomyocyte action potential duration (APD) has been shown to become prolonged via LPS (Robert et al., 2012), in this study, animals and animal cells were differenced, but isolated animal cardiomyocytes, and HL‐1, which are not ideal for mimicking human cardiac diseases. This fallacy is due to the inability to generate action potentials (APs) and contractions. Furthermore, cardiomyocytes from the human heart are virtually unavailable due to technical hurdles and safety concerns. Recently, human embryonic stem cell‐differentiated cardiomyocytes (HIPSC‐CMs) have demonstrated electrophysiological and pharmacological properties that are similar to those of native cardiomyocytes, including APs and responses to antiarrhythmic drugs (Germanguz et al., 2011).

In this study, we used human embryonic stem cell‐based cardiomyocytes and investigated the effect of LPS on arrhythmia, AP, sodium channel current, L‐type calcium channel current, and *I_k_* channel currents. Results from this investigation provide a potential mechanism of endotoxemia‐induced arrhythmia and suggest a theoretical basis of clinical treatment.

## 2 METHODS

### 2.1 Ethics statement

All of the procedures conformed to the NIH guidelines and were conducted following the protocols approved by the Animal Care Committee of Inner Mongolia University for Nationalities.

### 2.2 Generation of HIPSC‐CMs

Human embryonic stem cells were cultured and differentiated into HIPSC‐CMs according to previous research (Lu et al., 2014). The human embryonic stem cells were seeded into low adhesion six‐well plates in suspension culture to form embryoid bodies. After 7 days, the embryogenic bodies were inoculated into four‐well plates and adhered to the wells with the final concentration of 10 µmol/L 5‐azacytidine as differentiation induction. HIPSC‐CMs were kept at 37°C in a water vapor‐saturated atmosphere with 5% CO_2,_ and they were used from 24 hr up to 6 weeks after passaging with a medium change every 48 hr. The HIPSC‐CMs were divided into the following groups: the control group and the LPS group (0.04, 0.2, 1, and 5 ug/ml).

For whole cell patch clamp experiments, HIPSC‐CMs were incubated with collagenase CLSI for 30 min at 37°C, and then added to an Roswell Park Memorial Institute (RPMI) medium containing 10 FCS (Worthington, Germany) to collect HIPSC‐CMs.

### 2.3 Whole cell patch clamp experiments

The AP and ion channel currents were measured by standard patch clamp recording. Digidata 1440A, multiclamp 700B, micromotor drawing instrument MODEL P‐97, and three‐dimensional manipulator MP‐225 (Sutter Medical Technologies, Freiburg, Germany) were used for this study.

Patch electrodes were pulled from borosilicate glass capillaries using a DMZ‐Universal Puller. Patch electrodes were filled with electrode liquid. Before a Giga‐seal was formed, electrode offset potentials were zero‐adjusted. Fast capacitance was first compensated, and then the membrane under the pipette tip was disrupted by negative pressure after a Giga‐seal was generated to establish the whole cell configuration. The potential was recorded in a current‐clamp mode, and ion currents were analyzed in the voltage‐clamp mode.

The sodium channel current was recorded during depolarization, with the holding potential of −90 mV and a test potential −20 mV at room temperature. The current–voltage (*I–V*) curve of *I*_Na_ was obtained by plotting the *I*_Na_ peak current density at each test potential as the ordinate and the detection voltage as the abscissa. An external solution containing (mmol/L) choline chloride 110.0, NaCl 30.0, KCl 5.4, MgCl_2_ 1.0, HEPES 10.0, Glucose 10.0, tetraethylammonium (TEA) 10.0, 4‐AP 1.0, CdCl_2_ 0.2, and NiCl_2_ 0.1, pH 7.4 was used. The intracellular solution consisted of (mmol/L) CsCl 140.0, CaCl_2_ 1.0, MgCl_2_ 2.0, HEPES 10.0, EGTA 10.0, and Mg‐ATP 5.0, at pH 7.2.

The *I*_Ca‐L_ channel current was recorded during depolarization, maintained at a potential of −40 mV, to testing potentials +20 mV at a room temperature, and was then restored back to −40 mV. The recorded *I*_Ca‐L_ was fitted by the Boltzmann equation: *I*/*I*_max_ = 1 / {1 + exp [(*V* – *V*1 / 2) / *k*]}, where *I* and *I*_max_ are the current and maximum current at different test voltages Current, V denotes the pulse voltage, V1 / 2 denotes the pulse voltage of 50% of the channel inactivation, and k denotes the slope factor. An external solution containing (mmol/L) TEA 135, MgCl_2_ 0.53, CaCl_2_1.8, CsCl 20, and HEPES 5 at a pH of 7.4, and an intracellular solution consisting of (mmol/L) CsO H110, aspartic acid 90, CsCl 20, TEA‐Cl 10, HEPES 10, EGTA 10, Mg‐adenosine triphosphate (Mg‐ATP) 5, Na_2_ creatine phosphate 5, guanosine triphosphate (GTP) (Tris) 0.4, and leupeptin 0.1, at a pH of 7.2 was used.

The *I*_ks_ channel current was recorded during depolarization, from a holding potential of −80 mV to testing potentials 40 mV to −40 mV at room temperature. An external solution containing (mmol/L) 135 NaCl, 5 KCl, 1 MgCl_2_, 2.8 Na Acetate, and 10 HEPES, was used, and the pH was adjusted to 7.4 with NaOH. The intracellular pipette solution contained (mmol/L) 130 KCl, 5 EGTA, 1 MgCl_2_, 4 Na_2_‐ATP, 0.1 GTP, 10 HEPES, with the pH adjusted to 7.2 with KOH.

hERG K+ currents were recorded under a holding potential at −60 mV and then depolarized from −60 mV to 60 mV stepped by 10 mV for 4 s to activate the hERG K+ channel, then the peak tail currents were induced by a repolarizing pulse to −40 mV for 4 s. The bath solution contained 126 Choline‐Cl, 5 KCl, 2CaCl_2_, 1 MgCl_2_, 10 HEPES, and 5 glucose, with the pH adjusted to 7.4 with Choline‐OH. The solution was supplemented by 4‐AP (5 mmol/L), BaCl_2_ (0.5 mmol/L), CdCl_2_ (0.2 mmol/L), and chromanol 293B (10 μM) to suppress the potential interference of INa, Ito 1, IK1, ICaL, and IKs, respectively. A pipette solution containing 20 mmol/L KCl, 110 mmol/L K‐Aspartic, 1 mmol/L MgCl_2_, 5 mmol/L EGTA, 10 mmol/L HEPES, and 2 mmol/L Na_2_‐ATP was used, and the pH was adjusted to 7.2 with KOH.

### 2.4 Western blot analysis

Protein lysates were separated by Sodium Dodecyl Sulfate‐polyacrylamide gel electrophoresis, blotted onto polyvinylidene difluoride membranes, and incubated overnight with primary antibodies. The following concentration of primary antibodies was used (anti‐Kv7.1 1:200, anti‐Nav1.5 1:1000, anti‐Kv11.1 1:1000 [OriGene, Rockville, MD]; anti‐cTnT 1:1000, anti‐α‐actinin 1:1000, and anti‐calcium channel L‐type DHPR alpha 2 subunit 1:1000 Abcam, Cambridge, UK). Signals were detected and quantified by chemiluminescence. Glyceraldehyde‐3‐phosphate dehydrogenase (GAPDH) was used as the loading control.

### 2.5 Statistical analysis

All of the values are expressed as mean ± standard error of the mean. SSPS 17.0 statistical analysis software was used for data analysis. A paired Student’s *t* test was used to compare the different groups. The number of stars (*/#) indicate the *p* value range: *p value < 0.05, **p value < 0.01, ***p value < 0.001; ^#^ *p* < 0.05, ^##^ *p* < 0.01, and ^###^ *p* < 0.001.

## 3 RESULTS

### 3.1 LPS induced tachycardia in HIPSC‐CMs

**Figure 1:**
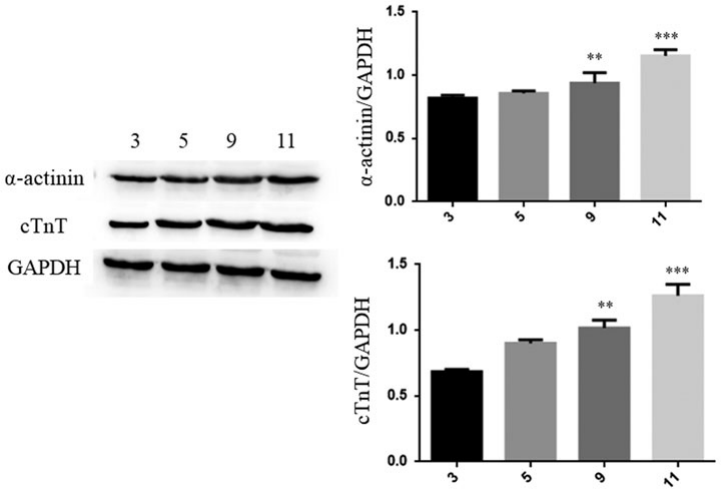
The characteristics of HIPSC‐CMs were analyzed by cardiac‐specific proteins. (a) Expression of cTnT in HIPSC‐CMs was assessed by western blot analysis. (b) Western blot analysis analysis of α‐actinin expression in HIPSC‐CMs. **p < 0.01 vs Con. cTnT: cardiac troponin‐T; HIPSC‐CMs: human embryonic stem cell‐differentiated cardiomyocytes; LPS: lipopolysaccharide

**Figure 2:**
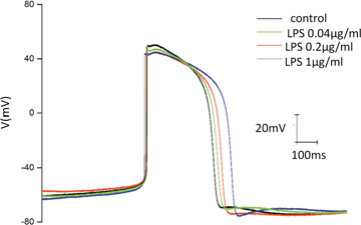
Effect of LPS on the spontaneous AP of HIPSC‐CMs. AP: action potential; HIPSC‐CMs: human embryonic stem cell‐differentiated cardiomyocytes; LPS: lipopolysaccharide [Color figure can be viewed at wileyonlinelibrary.com]

**Figure 3:**
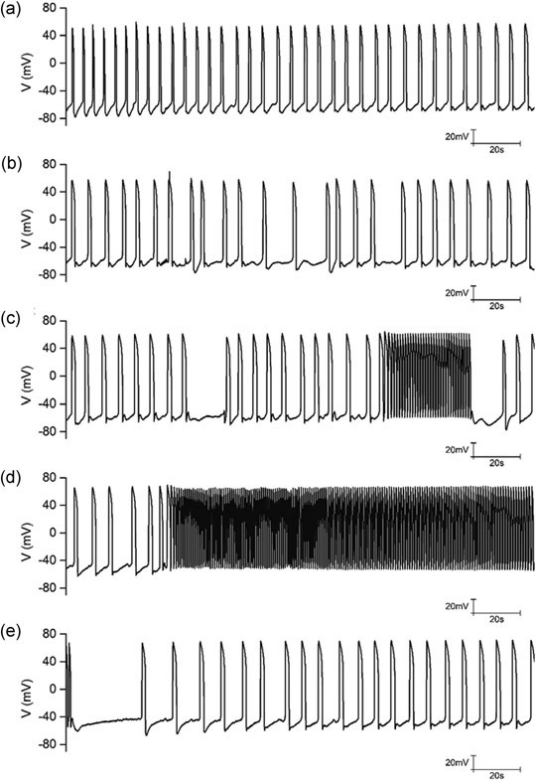
Effect of LPS (1 μg/ml) on the spontaneous AP of HIPSC‐CMs. (a) Representative recording showing the spontaneous AP before LPS application. (b) Decreased beating rate and prolonged AP duration after LPS (1 μg/ml) application. (c) Recovery from tachycardia after washout of LPS. Spontaneous AP under current clamp configuration and the membrane current was held at 0 mV. AP: action potential; HIPSC‐CMs: human embryonic stem cell‐differentiated cardiomyocytes; LPS: lipopolysaccharide

HIPSC‐CMs were analyzed by their characteristic cardiac‐specific proteins and AP traces. There was low expression of cardiac troponin‐T (cTnT) and α‐actinin, whereas the expressions of cTnT and α‐actinin were significantly increased on the ninth day of differentiation (Figure 1). It showed the typical AP trace of HIPSC‐CMs paced. The different doses (0,04, 0.2, and 1 μg/ml) of LPS were used to treat HIPSC‐CMs to evaluate the effects of LPS on the AP morphology. A significant difference was observed in APD among the control and LPS (0.04, 0.2, and 1 μg/ml) groups. LPS‐treated HIPSC‐CMs showed a dose‐dependent lengthening of the APD when compared with the control (Figure 2). Moreover, to analyze the effect of LPS on the spontaneous AP, HIPSC‐CMs were treated with 1 μg/ml LPS. Compared with the control, LPS treatment induced tachycardia in HIPSC‐CMs. Tachycardia dissipated after the washing of LPS (Figure 3).

### 3.2 LPS reduced L‐type calcium current in HIPSC‐CMs

**Figure 4:**
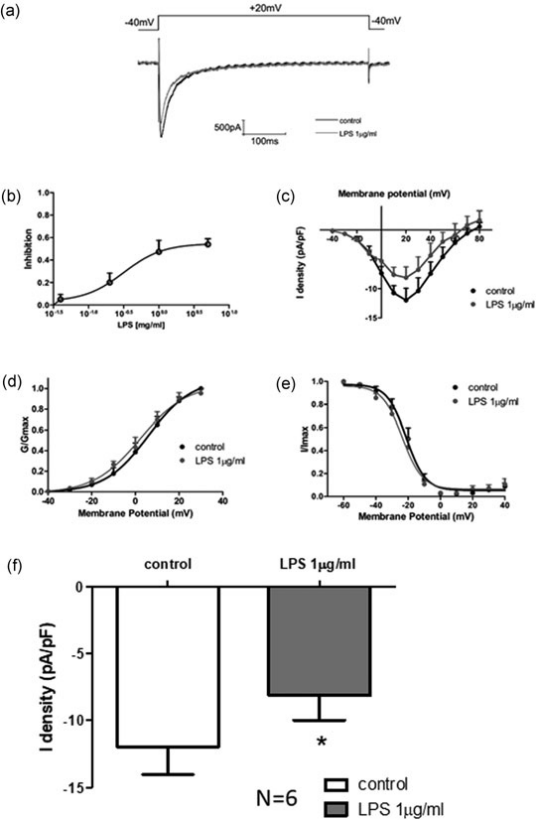
Effect of LPS on L‐type calcium channel currents in HIPSC‐CMs. (a) Representative traces of *I*_Ca‐L_ at +20 mV. (b) The inhibition of the *I*_Ca‐L_ current was analyzed by different doses of LPS. (c) The IC50 of *I*_Ca‐L_ was analyzed. (d) Representative activation curves of peak *I*_Ca‐L_. (e) Representative inactivation curves of peak *I*_Ca‐L_. (f) Mean values of Ca peak current at +20 mV. *p < 0.05 vs Con. HIPSC‐CMs: human embryonic stem cell‐differentiated cardiomyocytes; LPS: lipopolysaccharide

To investigate the ion channel currents that may contribute to the LPS‐induced change of APD, L‐type calcium current was analyzed in HIPSC‐CMs. LPS demonstrated weaker state‐dependent inhibition of the sodium channel current while dose‐dependently inhibiting inactivated L‐type calcium channel current (Figure 4a) with an IC50 of 1.254 µg/ml (Figure 4). *An I–V* curve was made with pulse voltage as abscissa, corresponding to the current density (pF/pA) as the vertical axis. LPS significantly inhibited *I*_Ca‐L_ current amplitude (pA/pF), demonstrating a 41% decrease in the peak current, but the *I*_Ca‐L_ voltage‐dependent activation and steady‐state inactivation were not statistically significant (Figure 4).

### 3.3 LPS reduced sodium channel currents (*I*_Na_) in HIPSC‐CMs

**Figure 5:**
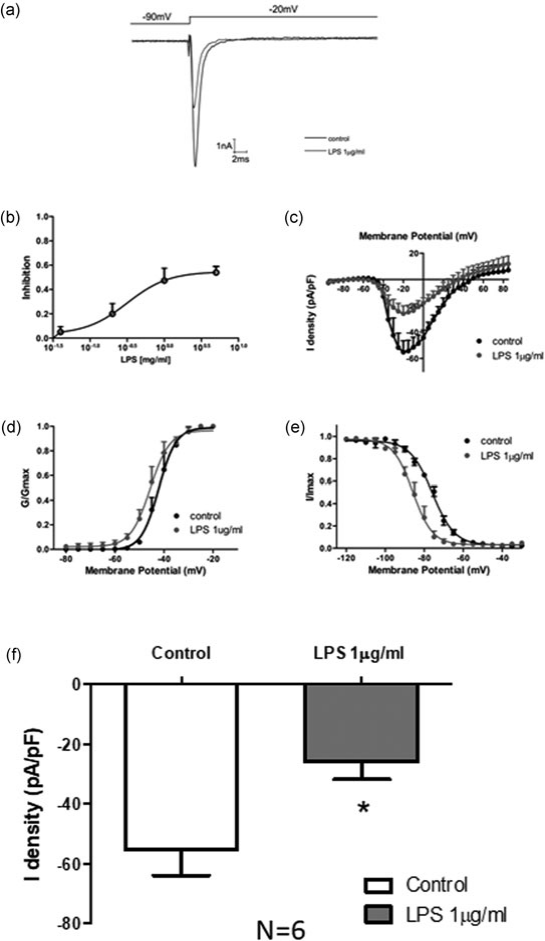
Effect of LPS on sodium channel currents in HIPSC‐CMs. (a) Representative traces of *I*_Na_ at −20 mV in the absence and presence of 20 µM TTX. (b) The inhibition of sodium channel currents was analyzed by different doses of LPS. (c) The IC50 of the sodium channel currents was analyzed. (d) Representative activation curves of peak *I*_Na_. (e) Representative inactivation curves of peak *I*_Na_. (f) Mean values of Na at –20 mV. *p < 0.05 vs Con. HIPSC‐CMs: human embryonic stem cell‐differentiated cardiomyocytes; LPS: lipopolysaccharide; TTX: Tetrodotoxin

The effect of LPS in prolonging the APD suggested that sodium channel currents (*I*_Na_) might be affected by LPS. The effects of LPS on *I*_Na_ were examined. LPS demonstrated strong state‐dependent inhibition of sodium channel currents while dose‐dependently inhibiting inactivated sodium channel currents (Figure 5a) with an IC50 of 1.254 µg/ml (Figure 5).

Compared with the control group, LPS decreased the amplitude of *I*_Na_ at all of the tested potentials; all five doses of LPS significantly reduced the inward peak amplitude of *I*_Na_ and *I*_Na_ current density, with a 61% decrease in the peak current. Next, the voltage‐dependent activation and steady‐state inactivation were evaluated by plotting Na^+^ peak current. The voltage‐dependent activation curve of *I*_Na_ was shifted left in HIPSC‐CMs with LPS (Figure 5d), and the steady‐state inactivation curve of *I*_Na_ in HIPSC‐CMs with LPS was shifted left (Figure 5e).

### 3.4 LPS reduced *I_k_* channel currents in HIPSC‐CMs

**Figure 6:**
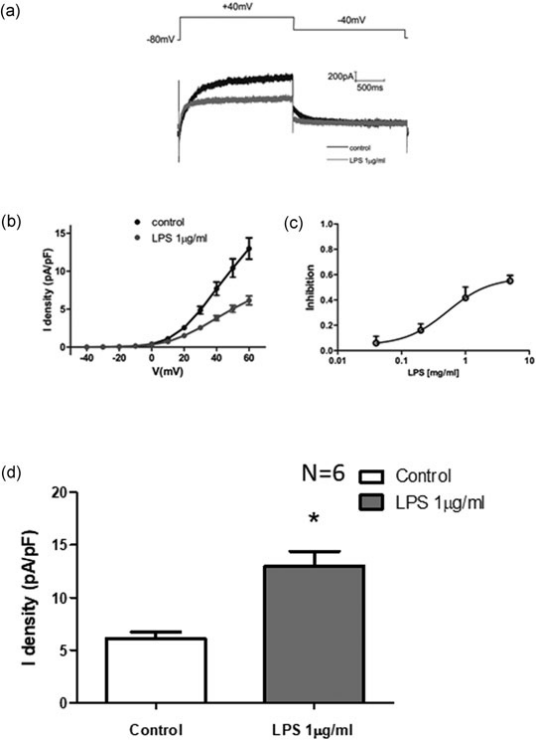
Effect of LPS on slowly delayed rectifier currents (*I*_Ks_) in HIPSC‐CMs. (a) The *I*_ks_ channel current was recorded during depolarization, from a holding potential of −80 mV to testing potentials 40 mV to −40 mV at a room temperature. (b) Mean values of *I*_Ks_ at +40 mV. (c) The IC50 of *I*_Ks_ was analyzed. (d) Mean values of *I*_Ks_ at +60 mV. *p < 0.05 vs Con. HIPSC‐CMs: human embryonic stem cell‐differentiated cardiomyocytes; LPS: lipopolysaccharide

**Figure 7:**
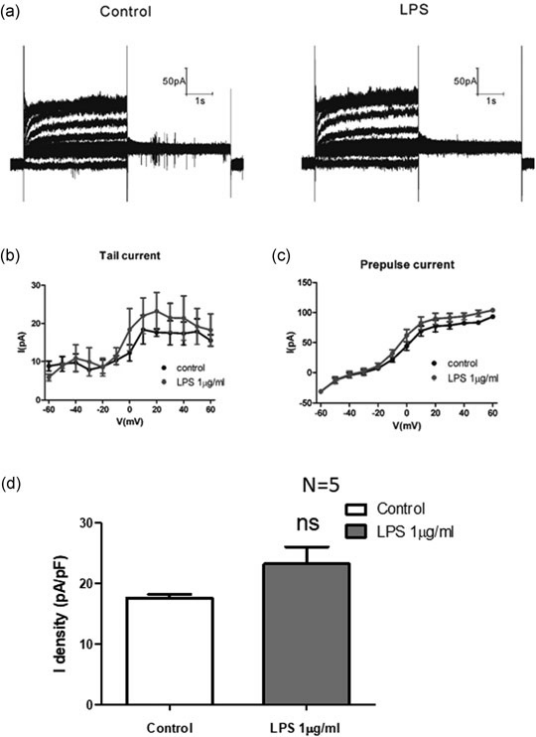
Effect of LPS on rapidly delayed rectifier currents (*I*_Kr_) in HIPSC‐CMs. (a and b) hERG K + currents were recorded under a holding potential at −60 mV and then depolarized from −60 mV to 60 mV stepped by 10 mV for 4 s to activate the hERG K + channel, then the peak tail currents were induced by a repolarizing pulse to −40 mV for 4 s. (c and d) The tail current and prepulse current of *I*_Kr_ were analyzed. (e) Mean values of hERG tail current (prepulse at +60 mV). HIPSC‐CMs: human embryonic stem cell‐differentiated cardiomyocytes; hERG: human ether‐a go‐go‐related gene; LPS: lipopolysaccharide

In human cardiomyocytes, outward rectifier potassium currents (*I*_k_) play a critical role in cardiac AP repolarization. *I*_k_ (*I*_ks_ and *I*_kr_) currents were activated and recorded. *I*_ks_ was quantified by fitting the Hill equation: *I*_compound_ / *I*_control_ = 1 /[1 + (the concentration of LPS / IC50)*^n^*], where IC50 is the drug concentration for 50% inhibition, and *n* is the Hill coefficient. Cultured HIPSC‐CMs were exposed to LPS at 0.04, 0.2, 1, and 5 μg/ml and *I*_ks_ was recorded 3 min later. The IC50 was 1.254 µg/ml. Furthermore, LPS shifted the half‐activation voltage of *I*_ks_ in HIPSC‐CMs with a 54% decrease in the peak current, and the slope factor significantly differed (Figure 6). LPS increased *I*_kr_ significantly (Figure 7).

### 3.5 LPS changed the expression of ion channels

**Figure 8:**
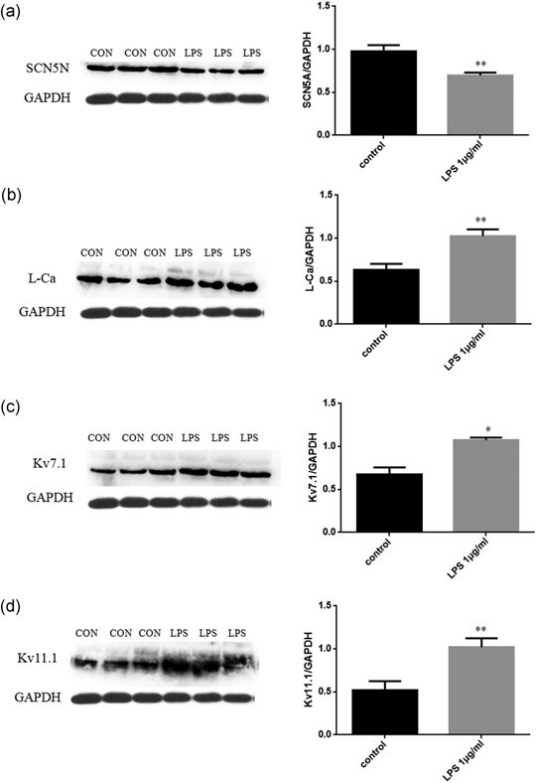
LPS induced changes in protein expression of ion channels. (a–d) The expression of Nav1.5/ICaL/Kv7.1 /Kv11.1 were analyzed by western blot analysis in HIPSC‐CMs after treatment by LPS. *p < 0.05, **p < 0.01 vs Con. HIPSC‐CMs: human embryonic stem cell‐differentiated cardiomyocytes; LPS: lipopolysaccharide

HIPSC‐CMs were treated for 24 hr with 1 μg/ml LPS. Protein expression of ion channels was analyzed by western blot analysis. LPS reduced the expression of Nav1.5 (Na^+^ channel, Figure 8a), but enhanced the expression of *I*_CaL_ (Figure 8b), Kv7.1 (*I*_Ks_, Figure 8c), and Kv11.1 (*I*_Kr_, Figure 8d).

## 4 DISCUSSION

This investigation demonstrates that LPS can modulate electrophysiological characteristics in HIPSC‐CMs. In our research, a longer APD and reduction of ion channel currents were identified in LPS‐treated HIPSC‐CMs. These results suggest that LPS plays a role in tachyarrhythmia, which may be the mechanism of endotoxemia‐induced human cardiac diseases.

As LPS is present in the outer cell membrane of gram‐negative bacteria, endotoxin was the principal mediator involved in the development of septic shock (Adamik et al., 2015). LPS was recognized by toll‐like receptor 4 in an immune cell, and then immune cells produced inflammatory responses, which led to the induction of a variety of processes that could contribute to the development of major organ failure (including heart) in the course of endotoxemia (Cunningham et al., 2002).

In this study, QT interval was enhanced, and a short array of arrhythmias occurred in HIPSC‐CMs induced by LPS, but this phenomenon disappeared after rupture (Figure 1). This is likely the cause of tachyarrhythmias in animal models and patients with inflammatory endotoxemia.

To clarify the electrophysiological mechanisms of dysfunction, the effects of LPS on the AP of cardiomyocytes were observed. The results showed that APD was prolonged (Figure 2). To identify the reason for the prolongation of APD, the ion channel currents were analyzed by whole cell patch clamp. Our study provides scientific evidence of cardiotoxicity of LPS at a low concentration as the IC50 (1.254 µg/ml) of LPS for suppressing *I*_k_ and *I*_Na_, but at a high concentration as the IC50 (5 µg/ml) of LPS for *I*_CaL_.

We found *I*_Na_ current density was reduced, and the voltage‐dependent activation curve of *I*_Na_ was shifted to the left in HIPSC‐CMs with LPS. In addition, the steady‐state inactivation curve of *I*_Na_ in HIPSC‐CMs with LPS was shifted left. These results suggest that endotoxin leads to low cardiomyocyte sodium channel abnormalities caused by gated dysfunction of the sodium channels, which may lead to the occurrence of cardiomyocyte conduction blockages. The activation and inactivation processes of sodium channels in cardiomyocytes were initiated at the same time and were triggered by the outward movement of the voltage‐sensitive S4 segment on the sodium channel currents. The cardiomyocytes were significantly slower than the activation process, so the zero‐period AP would continue to express introverted *I*_Na_. In this study, endotoxin changed the gaitral function of cardiomyocytes, and the activation and deactivation process likely leads to a decrease in the opening time of the sodium channels. A decrease in the opening time of the sodium channels can cause a decrease in *I*_Na_ sensitivity and a decrease in cardiomyocyte AP and membrane reactivity changes, leading to cardiac electrical excitability and electrical conduction abnormalities, and may even lead to Xi Shi Pu Karen system progressive conduction disorder, with or without left or right bundle branch block and QRS Wave widening. This effect could eventually cause a complete atrioventricular block, which may cause severe hemodynamic changes, resulting in syncope or sudden death in patients. Furthermore, *I*_k_ was inhibited in HIPSC‐CMs treated with LPS. As an important repolarization current, normal *I*_k_ could be in a quasi‐platform potential quasi‐equilibrium state to complete the normal complex polarization process. While the reduction of *I*_k_ would slow the repolarization of membrane potential, the formation of the platform phase quasi‐equilibrium potential and being prone to membrane potential shock led to arrhythmia. *I*_Ca‐L_ was inhibited in HIPSC‐CMs treated with LPS, which induced APD extension, and the ECG QT interval was prolonged, which triggered the early onset of arrhythmia. However, we did not observe significant changes of the activation curve and inactivation curve in HIPSC‐CMs treated with LPS.

In conclusion, this investigation identified the effect of endotoxin on the electrophysiology of human cardiomyocytes using HIPSC‐CMs. Our results demonstrated that the tachyarrhythmia of endotoxemia is involved with the prolongation of APD. The ion channel currents of *I*_Na_, *I*_CaL_, and *I*_k_ were decreased, which contributed to the extension of APD. These results provide a new theoretical basis and drug targets for the treatment of sepsis complicated with arrhythmia.

## FUNDING

This study was supported by the National Natural Science Foundation of China (No. 81360587, No. 81760780), the National Natural Science Foundation of Inner Mongolia (No. 2016BS0806), and the Mongolian Medicine Systems Biology Science and Technology Innovation Team Plan of Inner Mongolia.

## CONFLICTS OF INTEREST

The authors have no financial conflicts of interest.
